# Estimated Deaths and Illnesses Averted During Fungal Meningitis Outbreak Associated with Contaminated Steroid Injections, United States, 2012–2013

**DOI:** 10.3201/eid2106.141558

**Published:** 2015-06

**Authors:** Rachel M. Smith, Gordana Derado, Matthew Wise, Julie R. Harris, Tom Chiller, Martin I. Meltzer, Benjamin J. Park

**Affiliations:** Centers for Disease Control and Prevention, Atlanta, Georgia, USA

**Keywords:** Meningitis, fungal, disease outbreaks, disease modeling, fungi, deaths, illness, public health response, contaminated steroid injections, United States

## Abstract

During 2012–2013, the US Centers for Disease Control and Prevention and partners responded to a multistate outbreak of fungal infections linked to methylprednisolone acetate (MPA) injections produced by a compounding pharmacy. We evaluated the effects of public health actions on the scope of this outbreak. A comparison of 60-day case-fatality rates and clinical characteristics of patients given a diagnosis on or before October 4, the date the outbreak was widely publicized, with those of patients given a diagnosis after October 4 showed that an estimated 3,150 MPA injections, 153 cases of meningitis or stroke, and 124 deaths were averted. Compared with diagnosis after October 4, diagnosis on or before October 4 was significantly associated with a higher 60-day case-fatality rate (28% vs. 5%; p<0.0001). Aggressive public health action resulted in a substantially reduced estimated number of persons affected by this outbreak and improved survival of affected patients.

## Abstract 2

Public health response to the outbreak likely resulted fewer injections, cases, and deaths.

Deaths, Illnesses Averted in Meningitis Outbreak

In September 2012, the US Centers for Disease Control and Prevention (CDC), in collaboration with state and local health departments, initiated a multistate investigation into an outbreak of fungal infections linked to injections of preservative-free methylprednisolone acetate (MPA) produced at a single compounding pharmacy (New England Compounding Center [NECC], Framingham, MA, USA) (*1*,*2*). Three lots of MPA produced by NECC were implicated in this outbreak: 05212012@68, 06292012@26, and 08102012@51 (hereafter called lots 05, 06, and 08, respectively) (*2*). The Mycotic Diseases Branch Laboratory at CDC confirmed the presence of *Exserohilum rostratum,* an environmental mold and the primary pathogen in this outbreak, in lots 08 and 06 (*3*); detection of *E. rostratum* DNA in lot 05 was reported to CDC (*3*). This contamination resulted in one of the largest outbreaks of health care–associated infections and the largest outbreak of fungal meningitis documented in the United States. Thousands of public health officials at federal, state, and local levels, along with clinicians and administrative staff, worked over a period of many months to respond to this unprecedented outbreak.

CDC and partners quickly took action for several reasons: the high mortality rate seen in previous outbreaks of fungal meningitis (*4*,*5*), concern that subacute clinical signs and symptoms would not prompt exposed persons to seek health care evaluation until they had severe disease, and the large number of persons potentially exposed to contaminated MPA. Several key public health actions took place during the 10-day period of September 25–October 4, 2012. On September 25, NECC was informed that 3 MPA lots from its pharmacy appeared to be implicated in this outbreak. The next day, NECC issued a voluntary recall of the 3 lots. On September 28, CDC and state partners initiated efforts to notify all 13,534 persons potentially exposed to the implicated MPA to provide information on exposure and indications for seeking medical care.

CDC and partners developed diagnostic and treatment guidelines on the basis of expert opinion and incoming laboratory and patient data. These guidelines and subsequent updates were posted on CDC’s outbreak website (*6*) and disseminated through Health Advisory notices. A Health Advisory notice containing the first diagnostic and treatment guidance for this new clinical entity was distributed on October 3 (*7*) and was posted online on October 4. On that day, a joint Food and Drug Administration and CDC telebriefing (by telephone) publicized the outbreak and confirmed the presence of fungal contamination in unopened vials from lot 08 MPA (*8*). We conducted analyses to estimate the effect of these public health actions on the size and severity of this outbreak as measured by changes in the numbers of case-patients and deaths.

## Methods

### Overview of Modeling Used to Assess Effects of Public Health Actions

We restricted our analyses to patients with meningitis, including those with stroke caused by presumed meningitis. Cases of parameningeal (e.g., epidural abscess) or peripheral joint infection were not included in this analysis. To evaluate the effect of public health actions on the size of this outbreak, we sought to determine numbers of 1) MPA injections averted, 2) cases of meningitis or stroke averted, and 3) deaths caused by meningitis or stroke averted. Information about volume of MPA recalled was unavailable, so we estimated the recalled volume from data in NECC MPA shipping records. To estimate cases averted, we calculated attack rates in 2 steps. First, we modeled attack rates for patients with single injections. For patients with multiple injections, we conducted a Monte Carlo simulation that assigned a single responsible injection for each patient in each simulation. This simulation also provided estimates of uncertainty. To estimate deaths averted, we applied the case-fatality rate (CFR) for case-patients before most public health actions had occurred for the case-population at risk (i.e., number of case-patients diagnosed after October 4 and still alive 60 days later and estimated number of case-patients predicted to have occurred after October 4 if no recall had occurred).

### Case Definition

Case definitions developed during the outbreak response were used in this analysis (*9*). A case-patient was defined as a person who, after May 21, 2012, received an epidural or paraspinal MPA injection from an implicated lot and subsequently had either meningitis of unknown etiology or posterior circulation stroke without cardioembolic source and without a documented normal cerebrospinal fluid (CSF) profile. Meningitis was defined as >5 leukocytes/mm^3^ in CSF from a person with compatible symptoms (e.g., headache, stiff neck). Date of diagnosis was defined as the date when an initial lumbar puncture yielded CSF that met the meningitis case definition or the reported date of stroke diagnosis for stroke cases. Cases were defined as laboratory confirmed if histopathologic, microscopic, culture, or molecular evidence of a fungal pathogen was present and associated with the clinical syndrome. Inclusion in this analysis was limited to cases reported on or before the final case count published on October 23, 2013.

### MPA Injections Averted

NECC shipping records provided information on date of shipment, volume, and lot number of MPA shipped to each of the 75 injection facilities that received contaminated lots of MPA. To estimate volume of steroid recalled, we modeled the MPA use rate at each injection facility. For the 52 facilities that received >1 shipment of implicated MPA, we estimated a per-facility MPA use rate on the basis of reorder frequency and shipment volumes. This approach could not be used for 23 facilities that had a single shipment (i.e., they placed no reorders). For those facilities, we developed a simple linear regression model to estimate use rate with average volume of MPA as the predictor (Technical Appendix). We assumed that use of NECC MPA at each injection facility began on the first business day after a shipment date, that use occurred at the facility only on business days, and that use ceased completely on September 27, 2012. These methods enabled us to estimate weekly volume of NECC MPA used across all 75 clinics.

To estimate the volume recalled, we subtracted the total volume of estimated use before September 27 from the total volume shipped. To estimate the number of MPA exposures potentially averted, we divided the total volume of MPA recalled by 1 mL/injection volume (i.e., amount of MPA used per injection for 80% of patients). 

### Cases of Meningitis or Stroke Averted

Previously published data suggested that attack rates varied by lot number and age of the vial, with age being measured as length of time from vial production to injection date (*1*). Consequently, we anticipated that we would need to estimate separate attack rates for each of the 3 implicated MPA lots to account for vial age. To account for changes in attack rates by vial age, we calculated the attack rate for each calendar week during which injections took place and produced separate estimates for each lot of MPA. For case-patients with 1 injection, weekly lot-specific attack rates were calculated as follows: number of case-patients receiving an injection of lot X during a given week divided by estimated volume of lot X used that week (denominator was obtained from the MPA use rate analysis). A Poisson distribution was then fitted to the observed data to estimate the attack rate for each week during which injections were given (Technical Appendix). The model’s covariates were week of injection, lot number, and interaction between *week* and *lot* variables, selected because these variables showed evidence of modifying results. 

Next, for case-patients with >1 injection, we developed a probabilistic model that used a Monte Carlo simulation to randomly assign a single injection as responsible for each case-patient’s infection (Technical Appendix). A total of 100 simulations were performed; for each simulation, the probability of injection assignment was derived from the predicted weekly lot-specific attack rates among case-patients with only 1 injection. A Poisson regression model, using the same covariates as those used in the model for case-patients with 1 injection, was then fit to the 100 simulated datasets to produce a distribution of predicted number of cases (Technical Appendix). To capture additional uncertainty, 100 additional simulations of the predicted number of cases were generated so that 10,000 estimates of the predicted number of cases were generated for each week and lot combination. 

**Figure 1:**
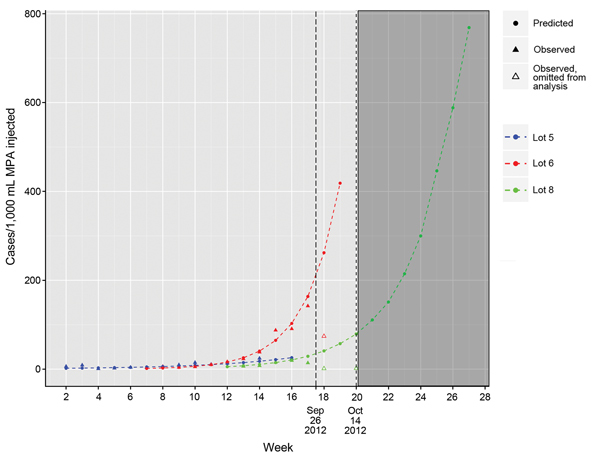
Weekly lot-specific observed and predicted attack rates (cases/1,000 mL injected) for illness among persons injected from 3 lots of methylprednisolone acetate (MPA) contaminated with the fungus *Exserohilum rostratum*, United States, 2012. Observed rates obtained by combining single injection data and 1 representative multiple injection simulated dataset (observed rates for multiple injections varied by simulation). Observations after September 23, 2012, were excluded in the prediction model. Long-dashed line indicates the date of the recall of the 3 lots of MPA (September 26, 2012); short-dashed line indicates the boundary of reliable estimates of attack rates in our model (October 14, 2012).

From this distribution, we then summed the median number of cases predicted for each week and lot combination after the recall date to obtain an estimated total number of cases averted. Finally, 95% CIs were calculated to capture uncertainty in our estimates (Technical Appendix). Extrapolation of weekly attack rates for lots 05 and 06 were continued until the model predicted that the volume of MPA in those lots had been completely used (Figure 1). However, lot 08 was in use for only 5 weeks before the recall on September 26, 2012. Thus, for this lot, we limited the extrapolation of attack rates and cases averted to 3 weeks after the recall (until October 14, 2012).

### Deaths and Strokes Due to Meningitis Averted

To estimate the number of deaths averted, we first calculated the 60-day CFR for patients given a diagnosis early in the outbreak (on or before October 4), before widespread publicity and posting of clinical treatment guidelines. This 60-day CFR was then applied to 2 groups of case-patients to estimate deaths averted: 1) patients given a diagnosis after October 4 who were still alive 60 days after diagnosis and 2) averted cases predicted to have occurred after October 4 without a recall.

### Evaluating Changes in Number of Deaths During the Outbreak

The 60-day CFR was plotted by week of diagnosis to examine mortality trends during the course of the outbreak. To understand whether these mortality trends were associated with changes in case-patient clinical characteristics, we compared measures of disease severity and treatment rapidity for case-patients given a diagnosis on or before October 4, 2012 to those given a diagnosis after that date. A χ^2^ test (dichotomous variables) and the Wilcoxon rank-sum (continuous variables) test were used. We estimated the survival function of time from diagnosis to death by using a Kaplan-Meier estimator. The log-rank test was used to compare survival of the 2 groups.

Data on case-patient clinical characteristics were collected from a standardized case report form created for this outbreak response. States reported case-patient deaths and dates of death routinely to CDC; thus, deaths occurring after completion of a case report form were captured systematically. The collection of patient-level data was deemed a public health emergency and was not subject to ethics review or informed consent procedures.

## Results

We identified 391 patients with meningitis or stroke caused by presumed meningitis in 19 states (*10*). Detailed data were available for analyses for 389 (99%) of these patients.

### Exposures Averted

NECC records indicated that 17,675 vials comprising 29,641 mL of the implicated MPA lots were distributed to 75 facilities in 23 states. Of the 11,773 mL of lot 05 shipped, we estimated that none was recalled; of the 10,847 mL of lot 06 shipped, we estimated that 132 mL (1%) was recalled; and of the 7,021 mL of lot 08 shipped, we estimated that 3,018 mL (43%) was recalled (Table 1) (Technical Appendix). A total estimated 3,150 mL of MPA was shipped but not administered because of the recall. Using an estimated per-injection volume of 1 mL, an additional 3,150 injections with the implicated lots of MPA could have occurred if all 3 lots of contaminated MPA had not been promptly recalled (Table 1).

**Table 1:** Methylprednisolone acetate (MPA) injections shipped, used, and recalled by lot*

Lot no.	Volume MPA shipped, mL	Volume MPA used, mL	Volume MPA recalled, mL
05	11,773	11,773	0
06	10,847	10,715	132
08	7,021	4,003	3,018
Total	29,641	26,491	3,150

*Volume of MPA shipped was derived from New England Compounding Center’s shipping records. Volume used and recalled was estimated by using the assumption that all shipped MPA was available for use at each clinic.

### Weekly Lot-Specific Attack Rates and Cases Averted

Of the 391 case-patients, 370 (95%) had injection data available for calculating attack rates. Of the 370 patients, 221 (60%) had 1 injection, 116 (31%) had 2 injections, 32 (9%) had 3 injections, and 1 (<1%) had 4 injections. Figure 1 shows observed and estimated weekly lot-specific attack rates in patients for each of the 3 contaminated lots of MPA.

For lot 05, predicted weekly attack rates increased from 1.9 (95% CI 0.8–4.7) cases/1,000 mL MPA administered during the first week of injections to 25.8 (95% CI 7.7–81.3) cases/1,000 mL MPA administered during the last week of injections; for these predictions, we assumed that injections continued until all vials were used. Lot 06 had predicted weekly attack rates that increased from 1.6 (95% CI 0.8–3.1) cases/1,000 mL MPA administered during the first week to 418.6 (95% CI 226.8–768.1) cases/1,000 mL MPA administered during the final week of injections (with the assumption that injections continued until all the vials were used). Lot 08 had predicted weekly attack rates that increased from 5.5 (95% CI 1.2–23.0) cases/1,000 mL MPA administered during the first week to 57.3 (95% CI 10.7–302.8) cases/1,000 mL MPA administered during the third week (October 14, 2012) after the recall notice. For all 3 lots, predicted attack rates rose as time from the medication production date increased (Figure 1). We estimate that without the recall of the 3 lots of MPA, 153 (95% CI 61–467) additional cases of meningitis would likely have occurred in persons exposed between September 26, 2012, and October 14, 2012 (Table 2).

**Table 2:** Meningitis and stroke cases and deaths averted by lot

Lot no.	Cases averted, no. (95% CI)	Deaths averted, no. (range)*
05	0	NA
06	53 (32–88)	NA
08	100 (29–379)	NA
Total	153 (61–467)	124 (98–211)**†**

*NA, not applicable. †Total deaths averted (124) derived from total cases averted (153) multiplied by the case fatality rate observed early in the outbreak (28%) plus case-patients who had a diagnosis of meningitis after October 4 and who were alive at 60 days (290) multiplied by the early case fatality rate: (153 × 0.28) + (290 × 0.28). Range derived from the upper and lower confidence intervals of cases averted (e.g., 98 = [61× 0.28] + [290 × 0.28]).

### Meningitis Deaths Averted

Of the 389 patients with available detailed data, 40 died <60 days of diagnosis (60-day CFR 10%). Of the 82 patients given a diagnosis on or before October, 23 (28%) died <60 days of diagnosis compared with 17 (6%) of the 307 patients given a diagnosis after October 4 (p<0.0001), an absolute risk reduction of 22% (Table 3). Of the 110 patients with laboratory-confirmed infection, 19 died <60 days of diagnosis (60-day CFR 17%). Of the 33 patients given a diagnosis on or before October 4, 13 (39%) died <60 days of diagnosis, compared with 6 (8%) of 77 who had a diagnosis after October 4 (p<0.0001) (Table 3).

**Table 3:** Clinical characteristics and 60-day case fatality rates for meningitis and stroke patients given a diagnosis on or before October 4, 2012, versus after October 4, 2012

Characteristic	Diagnosis date on or before October 4, n = 82	Diagnosis date after October 4, n = 307	p value
CSF parameters*			
Median leukocyte count, cells/μL	1,064	29	<0.0001
Median total protein, g/dL Median glucose, mg/dL	117 38	71 55	<0.0001 <0.0001
Median **no.** of symptoms	5	4	<0.0001
Antifungal treatment within 48 h of diagnosis	20 (59%)†	173 (84%)†	0.0006
Death within 60 d of diagnosis	23 (28%)‡	17 (6%)‡	<0.0001
Laboratory-confirmed cases only, n = 110	33	77	
Death within 60 d of diagnosis	13 (39%)§	6 (8%)§	<0.0001

*CSF, cerebrospinal fluid. †The denominators used for percentages represent the number of case-patients for whom data on antifungal treatment was available (34 and 206, respectively) for each diagnosis period.  ‡The denominators used for percentages are the total number of case-patients for each diagnosis period.  §The denominators used were the total laboratory-confirmed cases for each diagnosis period.

**Figure 2:**
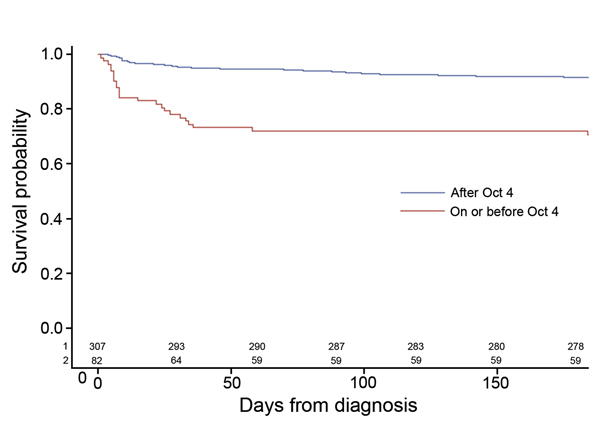
Kaplan-Meier (product limit) survival curves and persons at risk by date of diagnosis of meningitis and stroke case-patients among persons injected from 3 lots of methylprednisolone acetate contaminated with the fungus *Exserohilum rostratum*, United States, 2012. No patients were reported as lost to follow-up (e.g., censored) during the 6 months after their diagnosis. Values along horizontal axis indicate number of persons at risk by diagnosis date: 1) after October 4, 2012, or 2) on or before October 4.

**Figure 3:**
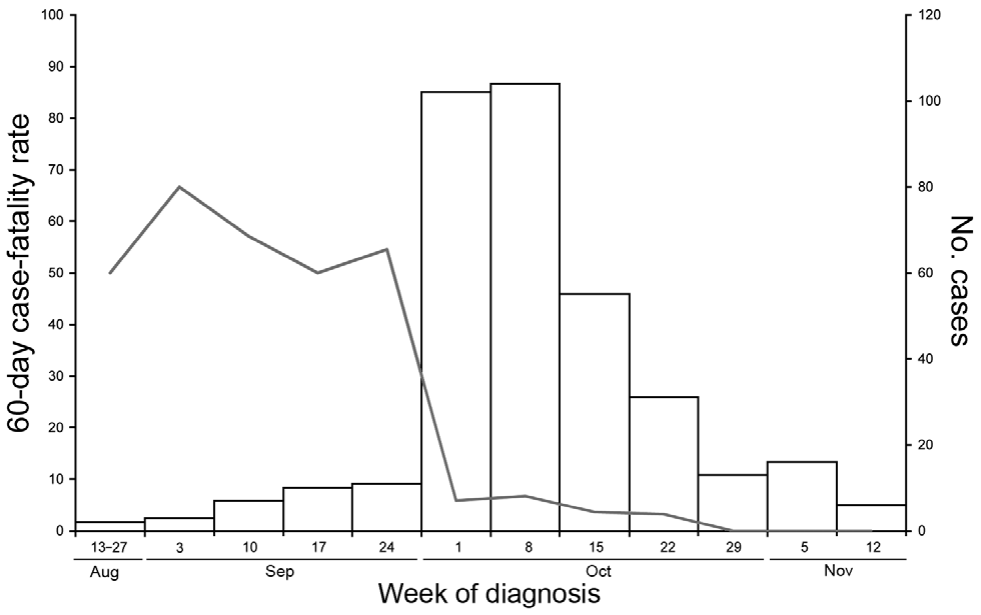
Sixty-day case-fatality rates (line) and case counts (bars) by week of diagnosis for meningitis case-patients among persons injected from 3 lots of methylprednisolone acetate contaminated with the fungus *Exserohilum rostratum*, United States, 2012.

Kaplan-Meier analysis and the log-rank tests showed that patients given a diagnosis after October 4 had improved overall survival compared with those given a diagnosis on or before October 4 (p<0.0001) (Figure 2). Patients given a diagnosis after October 4 also had lower CSF median leukocyte count (29 cells vs. 1,064 cells; p<0.0001); lower median CSF protein (71 g/dL vs. 117 g/dL; p<0.0001); higher median CSF glucose (55 mg/dL vs. 38 mg/dL; p<0.0001); and fewer symptoms when care was sought (median 4 vs. 5; p<0.0001) (Table 3). For the 240 patients with documented receipt of antifungal treatment, patients given a diagnosis after October 4 were more likely to receive antifungal drugs <48 hours of diagnosis (84% vs. 59%; p = 0.0006) (Table 3). The 60-day CFR by week of diagnosis was 50%–100% from August 27 through September 30; the CFR dropped substantially during the first week of October, to 3%–7% (Figure 3).

We applied the 60-day CFR (28%) of the 82 patients given a diagnosis of meningitis on or before October 4 to that of the 290 patients whose meningitis was diagnosed after October 4 and who were alive at 60 days (Table 3); we also applied that CFR to the 153 (95% CI 61–467) cases estimated to have been averted. With these calculations, we estimated that, without public health actions and early diagnosis and intervention by clinicians, 124 (range 98–211) additional deaths from meningitis or stroke may have occurred through October 14, 2012 (Table 2).

### Additional Use and Attack Rate Extrapolation

Extrapolation of the amount used from lot 08 suggests that, in the absence of a recall, its use would have continued until the week of December 2, 2012 (Figure 1). Estimates of attack rates and cases averted beyond October 14, 2012, are unreliable because of the short amount of time that lot 08 was in use before the recall. However, if lot 08 injections had continued through December 2 and if we assume an exponential rate of increase in attack rates (as was applied to lot 06), during the final week of injections, the attack rate for lot 08 might have been as high as 768.8 cases/1,000 persons exposed. Under these same assumptions, an additional 169 cases (total estimated cases = 322) and an additional 47 deaths (total estimated deaths = 171) would have occurred if lot 08 had been used through December 2. 

## Discussion

This outbreak of fungal meningitis and other infections triggered a public health response that involved thousands of public health officials, clinicians, and medical staff throughout the United States. This massive effort was commensurate with the scale of the public health crisis: a highly pathogenic fungus causing a clinical illness that had not been previously described and that initially resulted in a large number of deaths, with 13,534 persons potentially at risk. Despite the magnitude of this outbreak, we show that the effects of this outbreak, as measured by exposures, cases, and deaths, could have been far worse.

The 22% risk reduction in the CFR described here among case-patients given a diagnosis after October 4 was likely due to the direct effects of patient notification and clinician outreach, highlighted by intense activity of public health authorities during a 2-week period: multistate patient notification efforts were initiated (beginning September 28), a Health Advisory with diagnostic guidance was issued (October 4) (*7*), CDC’s first interim treatment guidance was developed and disseminated (October 4) (*6*), and the outbreak was publicized through a CDC–Food and Drug Administration joint telebriefing (October 4) (*8*) that publicly confirmed fungal contamination of MPA (*8*). Our analysis shows that these public health actions probably resulted in earlier diagnosis of infections at a less severe stage of disease and faster initiation of antifungal therapy; both actions may have contributed to a decreased number of deaths. The sharp decline in the 60-day CFR was probably not caused by inclusion of cases without fungal infection because the decline was also shown in laboratory-confirmed cases that had a CFR of ≈40% before October 4. 

The projected case estimates through October 14, 2012, are likely underestimates because we could not accurately extrapolate attack rates and cases averted because lot 08 was not used beyond this date. With no recall, injections with lot 08 would likely have continued for many weeks beyond October 14 (Figure 1), resulting in additional cases. We also assumed that no further contaminated lots were produced after production of lot 08. However, because 3 sequential lots of MPA were contaminated and serious sterility breaches were found during the onsite investigation at NECC (*11*), without a public health investigation and response, subsequent lots of contaminated MPA likely would have been produced, distributed, and administered. Ongoing contamination, leading to additional cases, would have occurred without rapid diagnosis and reporting of the index case (*12*), identification of the contaminated product, and subsequent public health actions. If we assume that subsequent MPA lots would be similar to the 06 lot in size, rate and amount of contamination, and attack rate, each additional contaminated MPA lot may have resulted in 275 additional cases of meningitis or stroke and 77 more deaths.

We found that the risk of meningitis and stroke was strongly influenced by the lot number and age of the MPA vial, with the latest produced lots (lots 06 and 08) and oldest vials conveying higher risk for disease than the earliest produced lot (lot 05) and younger vials of MPA (Figure 1). Because this MPA formulation lacked preservative, which inhibits fungal growth, fungus may have been better able to proliferate during extended storage times, possibly leading to high fungal contamination and attack rates. A study of compounded ophthalmic preparations reported increased fungal growth in preparations without common preservatives compared with preservative-containing solutions (*13*). Further studies are needed to confirm this observation for MPA. 

Several areas of uncertainty surround our estimates. First, data on lot exposure were not always recorded in patient charts. When these data were missing, available data, including clinic shipping invoices and manufacture and injection dates, were used to extrapolate the most likely lot exposure. Second, uncertainty surrounds our model for and estimates of recalled and unused MPA (*2*), and we did not carry the uncertainty surrounding the volume estimates forward in our subsequent modeling. We also assumed that all MPA use ceased the day after the recall notice and that clinics using NECC MPA did not use MPA from other manufacturers. We know that some clinics used non-NECC MPA and that a few clinics ceased use before the recall date, while others extended use beyond that date; these areas of uncertainty could have resulted in higher or lower estimates for MPA injections averted than are presented here. Other areas of uncertainty include the choice of the log link function used in the Poisson regression model, which has a progressively stronger effect on model predicted values outside the time range of the observed data. Finally, uncertainty exists in assigning responsible injections for patients with multiple procedures and in the parameter estimates in our Poisson modeling. A multiple simulation approach was used to capture uncertainty in these 2 areas and is expressed in the resulting ranges for our estimates of attack rates and cases averted. These multiple areas of uncertainty mean that the true number of injections, cases, and deaths averted might be higher or lower than our estimates.

This outbreak showed the devastating effect of contamination in a widely used product designated for sterile use. Public health actions, made possible by a strong existing public health infrastructure and rapid coordination among federal, state, and local partners, likely averted additional exposures, cases, and deaths. Maintaining this infrastructure and these partnerships is essential to preserve public health agencies’ abilities to respond quickly and meaningfully to future public health emergencies.

3. Centers for Disease Control and Prevention. Multistate outbreak of fungal meningitis and other infections—r**esources for laboratories**. 2014 [cited 2015 Mar 31]. http://www.cdc.gov/HAI/outbreaks/laboratory/index.html

**Technical Appendix.** Methods for modeling the use rate of New England Compounding Center Methylprednisolone Acetate.
