# Supplementary material for: Estimated Deaths and Illnesses Averted During Fungal Meningitis Outbreak Associated with Contaminated Steroid Injections, United States, 2012–2013
Source: Emerg Infect Dis. 2015 Jun;21(6):933–40. doi: 10.3201/eid2106.141558 (PMC4451895; doi:10.3201/eid2106.141558)
Supplement: Supplementary file 1 — Technical Appendix. Methods for modeling the use rate of New England Compounding Center Methylprednisolone Acetate. [file 14-1558-Techapp-s1.pdf]

Article DOI: http://dx.doi.org/10.3201/eid2106141558

Estimated Deaths and Illnesses Averted by Public Health Response to Fungal Meningitis Outbreak Associated with Contaminated Steroid Injections, 2012–2013, United States

Technical Appendix

Methods

Modeling the Use Rate of New England Compounding Center Methylprednisolone Acetate (MPA)

To estimate the use rate of New England Compounding Center MPA in the 22 facilities that ordered a single shipment of the implicated lots of MPA, we developed a simple linear regression model with errors assumed to be normally distributed with mean 0. The simple regression model used was

$$E(\text{burn rate}) = \beta_0 + \beta_1 * (\text{average volume used})$$

In this formula, "burn rate" = (burn volume)/(burn days) and "average volume used" = (burn volume)/(number of shipments – 1). "Burn volume" = total volume used between first and last shipment, "burn days" = number of days between first and last shipment.

The estimated model was

$$E(\text{burn rate}) = 0.84631 + 0.04692 * (\text{average volume used})$$

To assess the model fit for our simple linear regression, we considered the R^2 statistic. For our model, $R^2 = 0.57$, adjusted $R^2 = 0.56$. We also reviewed the residual plots and the normality plots (Figure 1). Review of these diagnostic plots suggests a reasonably strong fit.

Modeling Attack Rates for Case-Patients who Received Single Injections of MPA

We fitted a Poisson regression model to single-injection attack rate data. Observed single-injection data for outbreak weeks 17 and 18 (week of September 30 and October 7, 2012) were excluded from building the single-injection prediction model because they are based on data from a small number of laboratories that continued administering the contaminated injections after the recall; hence, the rates were much smaller for these 2 weeks. The model includes as covariates week of injection (*week*), lot number (*lot*), and an interaction between *week* and *lot* variables (denoted as *week**lot*). The *week*

variable was treated as a continuous variable. The Poisson regression model used was $\log \mu_{ij} = \beta_0 + \beta_1 * week_i + \beta_2 * lot_j + \beta_3 * week_i * lot_j + \log(V_{ij})$

In this model, V is the volume used per week; log(V) is the offset in the Poisson log-linear model; and $\beta_0 + \beta_1 * week_i + \beta_2 * lot_j + \beta_3 * week_i * lot_j + \log(V_{ij})$ is the *linear predictor*.

Parameters for this Poisson regression model can be found in Table 1. In this Poisson regression model, the data showed some indication of overdispersion (Table 2), and we used the scaled/adjusted standard errors to account for the overdispersion.

Modeling Attack Rates for Case-Patients who Received Multiple Injections of MPA

To assign case-patients with multiple injections of MPA to a single injection responsible for their infection, a method using Monte Carlo simulation was applied. We will illustrate the methods using the example of a case-patient who received 2 injections. Using the estimated attack rates from the single-injection model, we defined the probabilities of assignment for each of the 2 injections as follows:

Probability of injection 1: p1 = rate1/(rate1 + rate2)

Probability of injection 2: p2 = rate2/(rate1 + rate2)

We then used these 2 probabilities (note: p1 + p2 = 1) to define a discrete random variable and simulate random samples from a Bernoulli distribution with parameter p1. Methods for defining probability of assignment were similar for case-patients with 3or 4 injections. However, in cases of >2 injections, a generalization of the Bernoulli distribution for a categorical random variable (categorical or "table" distribution) was used (*1*).

This sampling was performed 100 times, and then each of the 100 simulated datasets was analyzed and had a Poisson model fit using the same covariates *lot*, *week*, and *week*lot*. Again, *week* was treated as a continuous variable. Once a Poisson regression model was fit to each of the 100 simulated datasets, the estimated model parameters (βs) with the corresponding standard errors (SE) were obtained. Figure 2 shows the distribution of the parameter estimates for each of the model parameters (*week*, *lot*, and *week*lot*) derived from the 100 simulated datasets. Lot 05 data are not presented as our estimates indicated that no volume of this lot remained at the time of the recall.

Next, we calculated the probability of a specific count outcome, the predicted number of cases. This calculation was done by using the parameter estimates for the 100 simulated datasets to obtain the estimated *linear predictor* of the Poisson model and the SE of the linear predictor. To capture the uncertainty surrounding the predicted number of cases, 100 "new" values of the *linear predictor* were simulated by adding to its estimate a randomly generated number *x* from the normal distribution with the

mean 0 and variance equal SE^2 ($x \sim N[0, SE^2]$). Thus, 10,000 estimates of the predicted number of cases were generated for each week and lot combination. Figure 3 shows the distribution of predicted number of cases for lot 6 during the week of September 30, 2012.

Cases Averted

Distributions of the predicted number of cases that would have occurred on weeks after the MPA was recalled were skewed. We used the median number of predicted cases averted for each week and lot combination. We then used the estimates between the 2.5th and 97.5th percentiles as the 95% Monte Carlo CIs.

Results

MPA Use

Clinics with 1 shipment of MPA were estimated to have a median daily use of 5.5 (range 1.3–94.7) mL of MPA. Clinics with >1 shipment were estimated to have a median daily use of 6.8 (range 0.4–9.9) mL of MPA.

References

1. Wicklin R. Statistical programming with SAS/IML software. Carey (NC): SAS Institute, 2010.

Technical Appendix Table 1. Parameter estimates from the Poisson regression model fit to single injection attack rate data

Parameter	Estimate	SE
Intercept	-6.6345	0.8664
Week no.	0.1540	0.1144
Lot 6	-3.8664	1.2926
Lot 8	-1.4996	4.1261
Lot 6 * week no.	0.3288	0.1345
Lot 8 * week no.	0.0816	0.2889

Technical Appendix Table 2. Goodness of fit statistic for the Poisson regression model fit to single-injection attack rate data*

Criterion	DF	Value	Value/DF
Deviance	16	24.3549	1.5222
Scaled deviance	16	12.7026	0.7939
Pearson χ^2	16	30.6770	1.9173
Scaled Pearson χ^2	16	16.0000	1.0000

*DF, Degrees of freedom

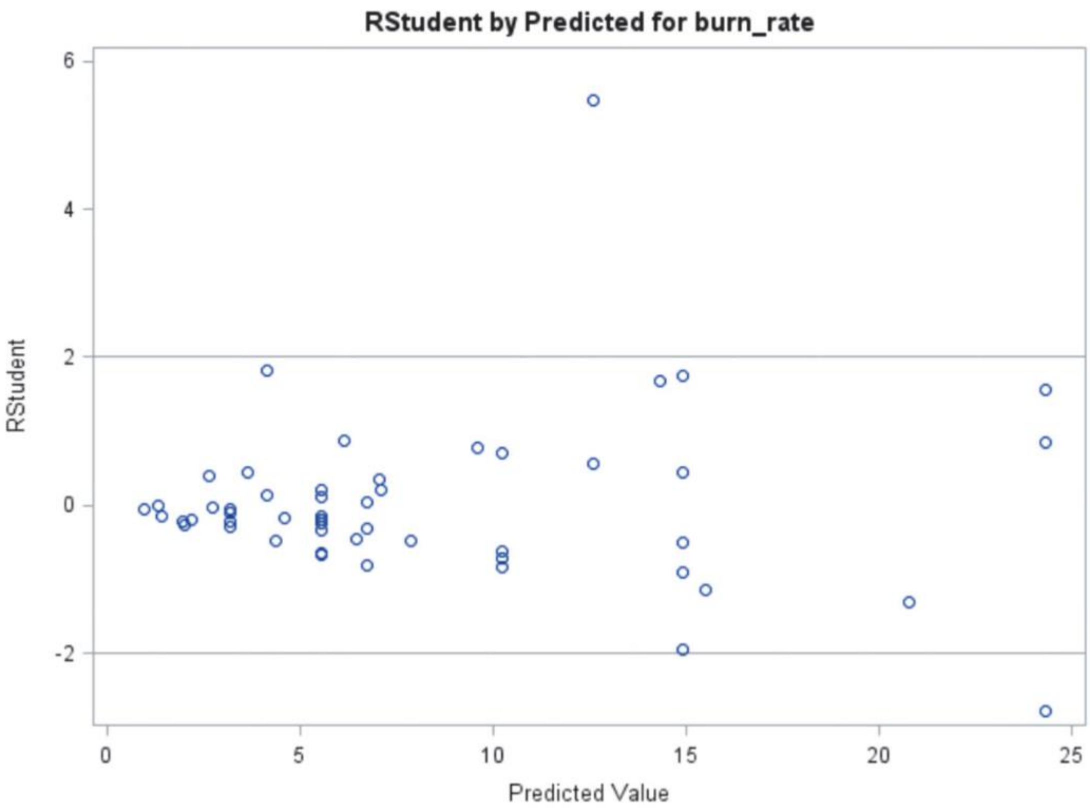

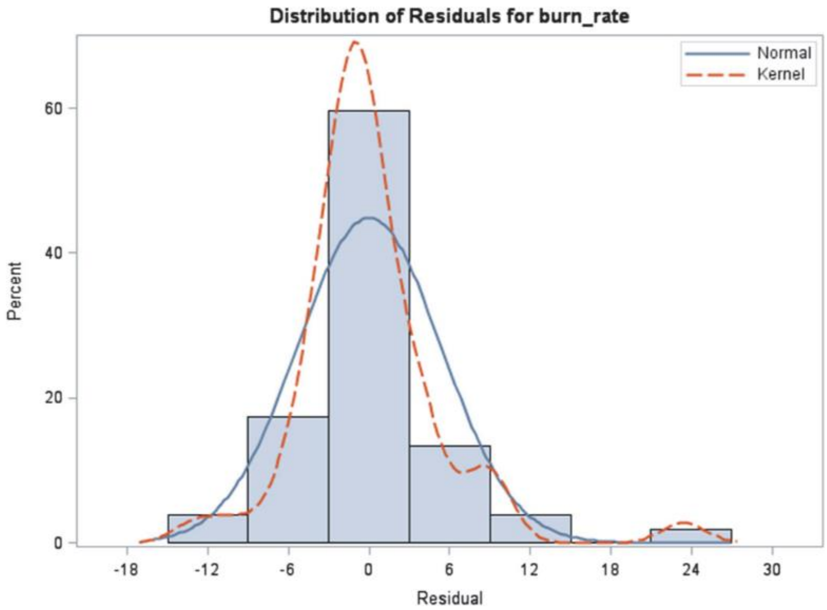

Technical Appendix Figure 1. Studentized residuals and normality plot for the linear regression model used to estimate use rate of methylprednisolone acetate.

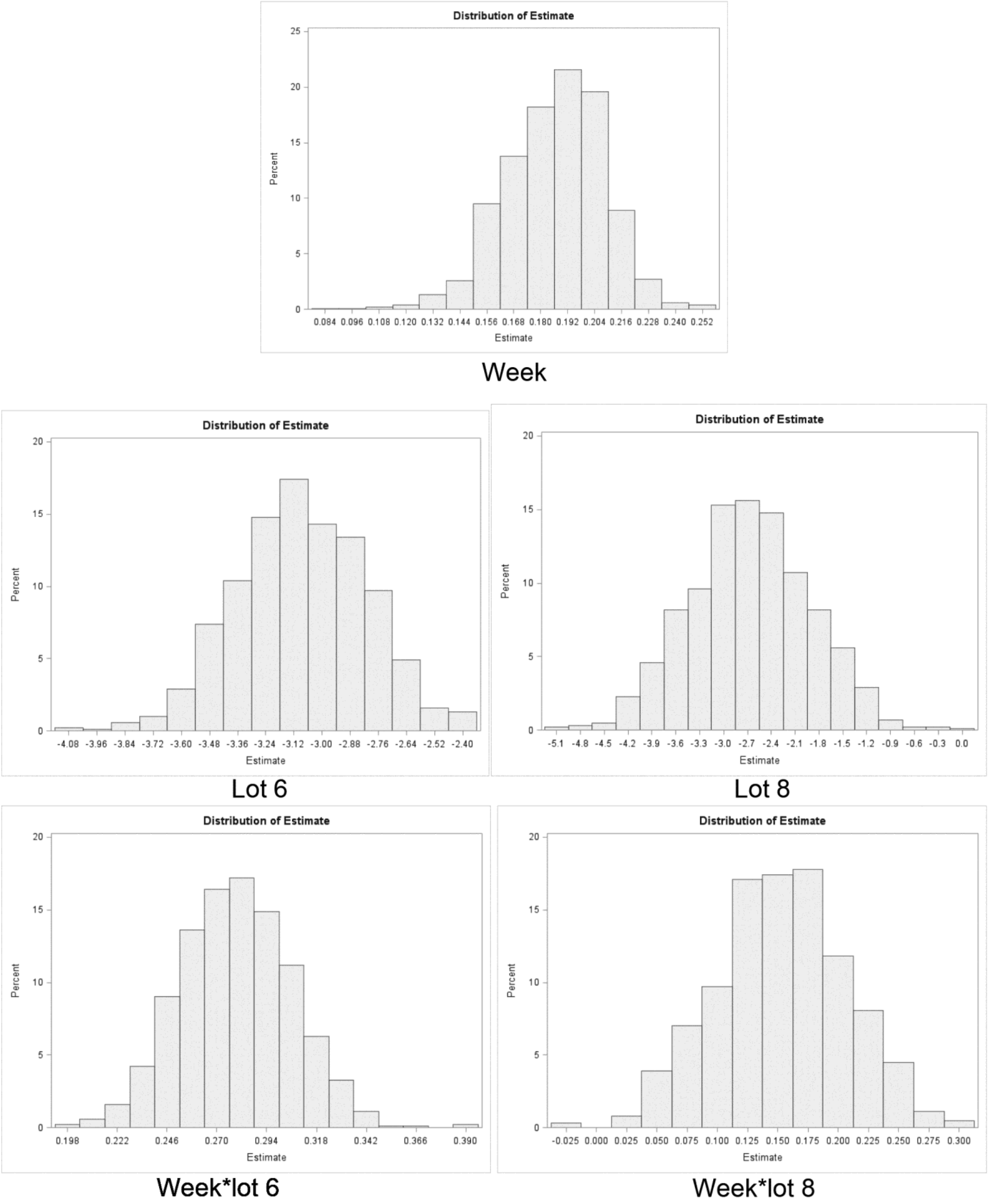

Technical Appendix Figure 2. Distribution of the parameter estimates for each of the model parameters (*week, lot,* and *week*lot)* on the basis of 100 simulated datasets. Lot 05 data is not presented because no residual volume remained at the time of the recall.

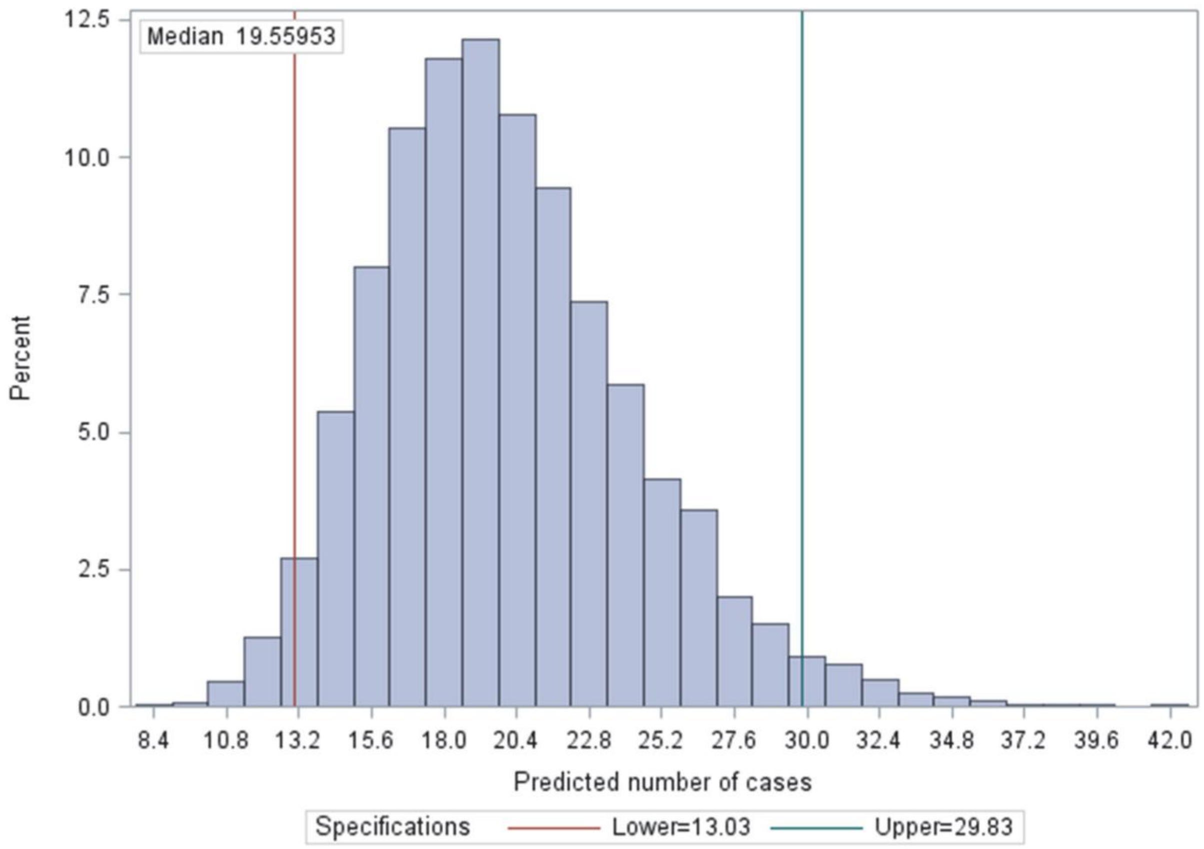

Technical Appendix Figure 3. Distribution of the estimated count for the week of September 30, 2012, for lot 06 after 10,000 simulations of the *linear predictor* with median, lower, and upper 95% CIs.
